# A common mechanism by which type 2A von Willebrand disease mutations enhance ADAMTS13 proteolysis revealed with a von Willebrand factor A2 domain FRET construct

**DOI:** 10.1371/journal.pone.0188405

**Published:** 2017-11-29

**Authors:** Christopher J. Lynch, Adam D. Cawte, Carolyn M. Millar, David Rueda, David A. Lane

**Affiliations:** 1 Department of Medicine, Centre for Haematology, Imperial College London, United Kingdom; 2 Department of Medicine, Molecular Virology, Imperial College, London, United Kingdom; 3 MRC London Institute of Medical Science, Single-Molecule Imaging Group, Imperial College, London, United Kingdom; 4 Imperial College Healthcare NHS Trust, Du Cane Road, London, United Kingdom; National Cerebral and Cardiovascular Center, JAPAN

## Abstract

Rheological forces in the blood trigger the unfolding of von Willebrand factor (VWF) and its A2 domain, exposing the scissile bond for proteolysis by ADAMTS13. Under quiescent conditions, the scissile bond is hidden by the folded structure due to the stabilisation provided by the structural specialisations of the VWF A2 domain, a vicinal disulphide bond, a calcium binding site and a N1574-glycan.The reduced circulating high MW multimers of VWF in patients with type 2A von Willebrand disease (VWD) may be associated with mutations within the VWF A2 domain and this is attributed to enhanced ADAMTS13 proteolysis. We investigated 11 VWF A2 domain variants identified in patients with type 2A VWD. In recombinant full-length VWF, enhanced ADAMTS13 proteolysis was detected for all of the expressed variants in the presence of urea-induced denaturation. A subset of the FLVWF variants displayed enhanced proteolysis in the absence of urea. The mechanism of enhancement was investigated using a novel VWF A2 domain FRET construct. In the absence of induced unfolding, 7/8 of the expressed mutants exhibited a disrupted domain fold, causing spatial separation of the N- and C- termini. Three of the type 2A mutants were not secreted when studied within the VWF A2 domain FRET construct. Urea denaturation revealed for all 8 secreted mutants reduced unfolding cooperativity and stability of the VWF A2 domain. As folding stability was progressively disrupted, proteolysis by ADAMTS13 increased. Due to the range of folding stabilities and wide distribution of VWF A2 domain mutations studied, we conclude that these mutations disrupt regulated folding of the VWF A2 domain. They enhance unfolding by inducing separation of N- and C-termini, thereby promoting a more open conformation that reveals its binding sites for ADAMTS13 and the scissile bond.

## Introduction

Von Willebrand factor (VWF) is one of the largest circulating proteins in the blood, due the multimerisation/concatemerisation which occurs during its biosynthesis. VWF expression occurs exclusively in endothelial cells and megakaryocytes, with the majority of VWF in the circulation derived from endothelial cell secretion. A mature VWF monomer is formed of domains in the order D’D3-A1-A2-A3-D4-C1-C2-C3-C4-C5-C6-CK[1], with multimerisation arising from disulphide bond formation between the CK to CK and D3 to D3 domains of neighbouring VWF monomers[2, 3].

Conformational changes in the VWF A1 and A2 domains of VWF underlie its propensity to interact with platelets and form a haemostatic plug. The large multimeric structure of VWF makes it susceptible to the rheological forces present in the vasculature, which influence the conformation of the individual domains[4]. Force is known to change the morphology of VWF from the globular native state of multimeric VWF to a more elongated conformation[5, 6], disrupting interdomain interactions[7]. A globular-to-stretch conformational change exposing the GPIBα site in the A1 domain precedes a subtle conformation shift in A1 required for its increased affinity for the platelet receptor GpIbα[8, 9], an initial step in platelet adhesion. More dramatic unfolding occurs in the VWF A2 domain, exposing binding sites for its regulating protease ADAMTS13 (a disintegrin and metalloproteinase with a thrombospondin type 1 motif, member 13) and its scissile bond (Y1605-M1606)[10]. ADAMTS13 proteolysis of the VWF A2 domain reduces the multimeric size of VWF.

The VWF A2 domain is structurally unique within VWF, being the only domain that lacks a domain spanning or multiple intradomain disulphide bonds:it does however contain a rare intradomain vicinal disulphide bond. The absence of the spanning disulphide bond provides the domain with flexibility, facilitating domain unfolding an essential requirement for ADAMTS13 proteolysis of VWF. The crystal structures of the VWF A2 domain[11–13] have guided biochemical and biophysical studies to understand domain flexibility, stability and unfolding. Three structural specialisations of the VWF A2 domain, a vicinal disulphide bond (C1669-C1670)[14], a calcium binding site (D1498, D1596, N1602)[12, 15] and a N-linked glycosylation (N1574)[16], have each been shown to provide folding stability. We have recently demonstrated how these structural specialisations of the VWF A2 domain cooperatively influence its stability[17] and that any reduction in stability enhances its interaction with ADAMTS13[16].

The regulation of force-dependent conformational changes is predicted to be disrupted by mutations identified in patients with qualitative defects of VWF as seen in type 2 von Willebrand disease (VWD). The loss of high molecular weight (HMW) VWF multimers in type 2A VWD may result from a variety of pathogenic mechanisms, with mutations located across multiple VWF domains[18–22]. Type 2A VWD variants have historically been classified according to whether they affect the intracellular assembly and secretion of VWF multimers (type 2A group 1) or the susceptibility to proteolysis by ADAMTS13 of VWF that is secreted normally (type 2A group 2)[18, 19]. The VWF A2 domain spans 175 amino acid residues (M1495-C1670) and over 30 missense mutations associated with type 2A VWD are clustered within this region[7, 23]. A recent study of mechanisms in type 2A VWD has shown that VWF gene (*VWF)* gene mutations, including those within the A2 domain, may display both VWF assembly/secretion defects and enhancement of ADAMTS13 proteolysis[24]. Biochemical and biophysical studies of purified recombinant fragments of VWF, containing selected type 2A VWD mutations within the VWF A2 domain, reveal that the mutations perturb the thermostability and unfolding of the VWF A2 domain[25] and enhance ADAMTS13 proteolysis of a VWF A1A2A3 domain fragment[26]. Molecular dynamic simulations in which VWD mutations were introduced into the VWF A2 structure, predict that the mutations will reduce the force required to unfold the VWF A2 domain[27–33].

For the presently reported work, we have developed a chimeric VWF A2 domain in which fluorescent proteins have been fused to the N- and C- termini of the VWF A2 domain. This enables monitoring of the primary function of the VWF A2 domain, regulated unfolding and proteolysis by ADAMTS13, by Förster/fluorescence energy resonance transfer (FRET). We show that a common feature of type 2A mutations located within the VWF A2 domain is the enhancement of VWF A2 domain unfolding, manifested by spatial separation of the N- and C-termini with reduced folding cooperativity and stability. As VWF A2 domain unfolding progresses, due to either urea and/or the type2A mutations, the susceptibility to proteolysis by ADAMTS13 is increased.

## Methods

### Selection of VWF variants

A variety of VWF A2 domain point mutations reported on the International Society on Thrombosis and Haemostasis-Scientific and Standardization Committee (ISTH-SSC) VWF database in association with type 2A VWD, (n = 10) were chosen to represent variants located across the domain (Table 1) (http://www.hemobase.com/vwf or http://www.vwf.group.shef.ac.uk/). Diagnostic laboratory values, where available, are reported for the mutations and expressed in iu/ml.

**Table 1:** VWD diagnostic laboratory values and recombinant expression data.

Mutation	Diagnostic laboratory data available				Recombinant FLVWF expression				
		VWF:Ag (iu/ml)	VWF:RCo (iu/ml)	FVIII:C (iu/ml)	Medium (ng/ml)		Lysate (ng/ml)		Ratio M:L
WT					600	±193	665	± 232	0.9
V1499E	yes[39]	0.22–0.59	<0.15–0.21	0.22–0.58	59.6	± 12.4	396	± 95	0.2
G1505E	yes[18]	0.62	n/a	n/a	427	± 142	489	± 6	0.9
G1505R	yes[18]	0.21	n/a	n/a	94.3	± 45.3	445	± 83.3	0.2
M1528V		n/a	n/a	n/a	524	± 271	594	± 236	0.9
L1540P	yes[40]	0.47	0,28	1.29	31.4	± 12.3	440	± 166	0.1
L1562P		n/a	n/a	n/a	515	± 149	491	± 120	1.0
R1597W†	yes[41]	0.28	0.09	0.29	579	± 226	605	± 213	1.0
V1604F		n/a	n/a	n/a	326	± 159	288	± 147	1.1
D1614G		n/a	n/a	n/a	791	± 158	705	± 78.0	1.1
G1629R‡	yes[42, 43]	0.31–1.0	0.04–0.30	0.41–0.91	881	± 514	579	± 254	1.5
D1653V		0.46	0.26	0.80	738	± 243	567	± 95.2	1.3

† R1597Q reported in VWD database

‡ G1629E studied[26] but not reported in VWD database

Results of recombinant FLVWF transient expression are means ±SD of at least 3 independent experiments. n/a not available.

A novel unreported D1653V variant, with diagnostic laboratory values, identified in a patient diagnosed with VWD type 2A was also included.

### Recombinant FLVWF protein expression and characterisation

Mutations were introduced into recombinant full-length VWF (FLVWF) and the protein transiently expressed in HEK293 cells (ThermoFisher #11631017), concentrated, quantified by ELISA and multimeric structure analysed by gel electrophoresis as previously described[16, 17].

### ADAMTS13 proteolysis of FLVWF

FLVWF and its variants (500ng/ml) and recombinant ADAMTS13 (10nM), expressed as previously described[16, 17] were separately pre-incubated with 20mM Tris (pH7.8), 150mM NaCl, 5mM CaCl_2_ ± 2M Urea for at 37°C for 45mins. Samples were combined and incubated at 37°C for proteolysis to occur. Reactions were terminated after 4 hours by the addition of EDTA. Multimer formation was analysed on a 2% agarose gel and VWF bands detected on a Western blot with a polyclonal anti-VWF antibody.

### Recombinant VWF A2 FRET construction, protein expression and characterisation

The VWF A2 FRET expression plasmids were generated using the PCEP-A2VicCC[14, 16, 17], pcDNA3-Clover[34] and pcDNA3-mRuby2[34] expression plasmids as templates (pcDNA3-Clover and pcDNA3-mRuby2 were kind gifts from Michael Lin, Addgene plasmids #40259 and #40260). Primers were designed using the NEBuilder® assembly tool; subsequent PCR-generated fragments were purified using a gel extraction Kit (Qiagen #28704) and vectors assembled using the NEBuilder® HiFi DNA assembly kit. In brief, a large fragment was PCR amplified from PCEP-A2VicCC (with appropriate overhangs) containing PCEP backbone with C-terminal MycHis-tag and N-terminal VWF-signal peptide followed by VWF residues M1472-P1490. The mRuby2 sequence (V2-K237) was PCR amplified from pcDNA3-mRuby2 (with appropriate overhangs) and inserted C-terminal to the VWF P1490 sequence of the large PCEP-A2VicCC fragment. A smaller fragment was PCR amplified from PCEP-A2VicCC (with appropriate overhangs) and contained the VWF A2 domain sequence P1490-G1672, and was inserted C-terminal to the mRuby2 sequence. Finally, the clover sequence (V2-K239) was PCR amplified from pcDNA3-clover (with appropriate overhangs), inserted C-terminal to the VWF A2 sequence and N-terminal to the MycHis-tag sequence, completing the circular vector. The new circular expression vector was termed PCEP-A2F-R-A2-C (protein termed R-A2-C) as mRuby2 was N-terminal to the VWF A2 sequence and clover was C-terminal. The PCEP-A2F-C-A2-R expression plasmid was generated in a similar manner(protein termed C-A2-R). Primers to generate the deletion variants (PCEP-A2F-A2-C, PCEP-A2F-R-A2, PCEP-A2F-C-A2, PCEP-A2F-A2-R) and PCEP-A2F-C-R expression plasmids (where the VWF A2 domain was truncated to a six amino acid sequence, PKRNSG) were designed using the NEBasechanger® tool and assembled using the NEB Q5® Site-Directed Mutagenesis Kit (all primer, vector sequences and plasmids available on request). The VWF A2 FRET variants were transiently transfected in HEK293 EBNA cells[35], purified by Ni^2+^ affinity as monomeric proteins and quantified as previously described for PCEP-A2VicCC[14, 16, 17]. The recombinant VWF A1A2A3 fragment (residues 1260–1873 with a C-terminal myc/his tag) was expressed and purified as described above.

### SDS-PAGE and Western blot analysis of VWF A2 FRET samples

Protein samples were boiled in loading buffer (Invitrogen) before being run on a 4–12% SDS-PAGE gel. Samples were either directly stained with Coomassie or transferred to a nitrocellulose membrane which was incubated for 1 hour in blocking solution (3% BSA and 5% milk PBS 0.1% Tween-20). Transferred proteins were detected by incubating the membrane with a mouse anti-myc-HRP antibody. The membrane was washed with PBS 0.1% Tween-20 and then incubated with a chemiluminescent HRP substrate and exposed to film.

### FRET measurements of VWF A2 FRET proteins

Bulk FRET measurements of the clover/mRuby2 tagged VWF A2 fragments were performed using a Cary Eclipse Fluorescence Spectrophotometer (Agilent Technologies). Excitation at 505nm ±5nm was used for FRET measurements and band pass filters for the excitation and emission were 335-620nm and 430-1100nm, respectively. A constant temperature of 37°C was used for all scans and controlled by a Cary temperature controller (Varian). Measurements were made using quartz cuvettes (Starna Scientific #16.100-F/Q/10/Z15) containing a volume of 120–175μl of 50nM VWF A2 FRET constructs in 20mM Tris (pH7.8), 50mM NaCl, 1.25mM CaCl_2_ (unless otherwise stated in the text). The ‘observed FRET’ (FRET_obs_) was calculated from Ex_505_ emission scans using the equation: FRET_obs_ = Ex_505_ Em_585_ / (Ex_505_ Em_516_ + Ex_505_ Em_585_). The urea concentration of the mid-point of unfolding (K_urea_) and cooperativity of unfolding (Hill coefficient, n) were calculated by fitting the urea titration data using a Hill titration function (Wavemetrics IGOR Pro): f(U) = FRET_max_-(FRET_max_-FRET_min_)*U^n/(k^n+U^n). Where K is the titration midpoint and n is the cooperativity coefficient and U the concentration of urea in M.

### Time course of ADAMTS13 proteolysis of VWF A2 FRET

The VWF A2 FRET variants and recombinant ADAMTS13[16, 17] were pre-incubated in 20mM Tris (pH7.8), 50mM NaCl, 1.25mM CaCl_2_ and 0–2.5M urea for at least 45 minutes before being combined. Initially, 120μl of VWF A2 FRET variants were added to the cuvette with a final concentration of 50nM and Ex_505_ emission scans taken every 0.5 minutes for 120 minutes. After 2 minutes, 55μl of ADAMTS13 was added to reach a final concentration of 50nM or 5nM. Reactions were terminated after 120 minutes by addition of EDTA, 80mM, for subsequent analysis by western blot. The FRET_obs_ for each 0.5 minute time point was calculated from the Ex_505_ emission scans and converted to ‘Fraction Cleaved’ (FC) using the equation: FC = (([F_obs_]_0_—FRET_min_)—([F_obs_]_t_-FRET_min_))/ ([F_obs_]_0_—FRET_min_). Where, [F_obs_]_0_ is the average FRET_obs_ value before the addition of ADAMTS13 (i.e. 0-2mins), FRET_min_ the calculated FRET_min_ value for each mutation (Table 2) and [F_obs_]_t_ the time point FRET_obs_ value. The FC time course data was fitted to an exponential rise function (Wavemetrics IGOR Pro), f(t) = FC_0_+FC_max_*(1-exp(-t/tau)), and half-lives (tau) calculated by holding FC_0_ to 0 and holding FC_max_ to 1 (when FC_max_<0.75).

**Table 2:** Quantitative values from urea denaturation of VWF A2 FRET variants.

	FRET_max_			FRET_min_			K_urea_ (M)			n		
**WT CaCl_2_**	0.20	±	0.01	0.08	±	0.01	2.16	±	0.13	7.1	±	3.0
**WT EDTA**	0.19	±	0.01	0.08	±	0.01	0.77	±	0.03	2.3	±	0.2
**PNG CaCl_2_**	0.18	±	0.01	0.08	±	0.01	1.47	±	0.10	3.0	±	0.4
**PNG EDTA**	0.14	±	0.01	0.07	±	0.01	1.11	±	0.24	1.1	±	0.2
**G1505E**	0.14	±	0.01	0.07	±	0.01	0.78	±	0.11	1.3	±	0.2
**M1528V**	0.19	±	0.01	0.08	±	0.01	1.21	±	0.05	3.7	±	0.5
**L1562P**	0.17	±	0.01	0.08	±	0.01	0.70	±	0.04	1.5	±	0.2
**R1597W**	0.16	±	0.01	0.08	±	0.01	1.22	±	0.16	1.5	±	0.3
**V1604F**	0.18	±	0.01	0.08	±	0.01	0.96	±	0.04	2.6	±	0.3
**D1614G**	0.19	±	0.01	0.08	±	0.01	0.95	±	0.04	2.2	±	0.2
**G1629R**	0.18	±	0.01	0.08	±	0.01	0.86	±	0.03	2.0	±	0.1
**D1653V**	0.20	±	0.01	0.09	±	0.01	1.32	±	0.06	2.9	±	0.3

K_urea_ = the concentration of urea at the mid-point of unfolding.

n = Hill coefficient of unfolding.

Results are means ±SD of at least 3 independent experiments.

## Results

### FLVWF expression, multimer structure and ADAMTS13 proteolysis

WT FLVWF transiently transfected into HEK293 cells displayed a media to lysate (M:L) ratio of ~1, indicative of the regulated storage and secretion of VWF from the pseudo-WPBs formed in HEK cells[36–38]. Of the 11 VWF A2 variants, eight displayed comparable secretion to that of WT FLVWF (Table 1). However, 3 of the 11 variants had very low secretion, with a M:L ratio of <0.25, consistent with a type 2A group 1 VWD mechanism (Table 1).

**Fig 1:**
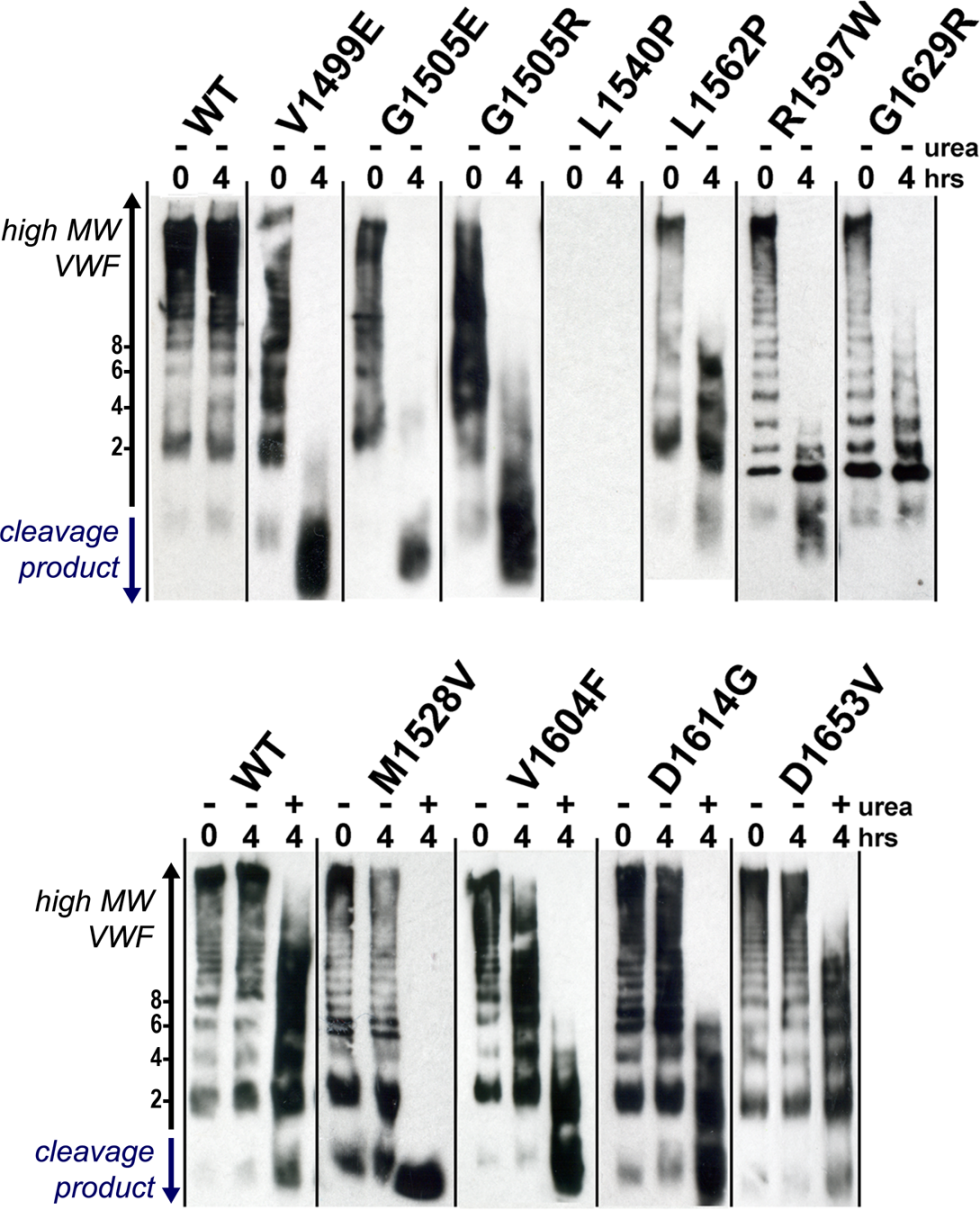
Multimer structure and ADAMTS13 proteolysis of FLVWF variants. The multimeric structures of recombinant FLVWF and its variants (500ng/ml) (with the exception of L1540P where no protein was expressed), were analysed before (0hrs) and after a four hour incubation with ADAMTS13 (10nM) (4hrs) in the presence (+) or absence (-) of 2M urea. The number of VWF monomers within a band and the direction of increasing multimeric size are indicated in black. Upon ADAMTS13 proteolysis, high MW VWF is lost, and cleavage products appear below the dimeric (2) band.

Except for the L1540P variant, where VWF levels were so low no protein could be detected, the majority of variants displayed a full range of VWF multimers, similar to that observed for WT FLVWF (Fig 1). The V1499E, G1505E/R and L1562P variants may have been somewhat more prone to degradation by intracellular/extracellular proteases, as smearing of VWF bands can be observed at time point 0, something that we have previously observed with destabilised FLVWF variants [17]. After a 4-hour incubation with ADAMTS13 (in the absence of denaturant) a reduction in VWF multimer size could be detected for 6 of the variants, indicative of proteolysis by ADAMTS13 (Fig 1, upper panel). Full proteolysis of VWF was detected for the V1499E, G1505E, G1505R and R1597W, with the multimeric structure completely ablated. Partial proteolysis of the L1562P and G1629R variants occurred, indicated by the loss of only higher MW multimers. The WT, M1528V, V1604F, D1614G and D1653V variants displayed minimal to no evidence of ADAMTS13 proteolysis under these conditions.

ADAMTS13 proteolysis assays were repeated in the presence of urea for these 4 uncleaved variants. After a 4 hour incubation with ADAMTS13 in the presence of 2M urea, a notable loss in multimer structure could now be observed for M1528V, V1604F and D1614G (Fig 1, lower panel). The WT and D1653V variant displayed resistance to ADAMTS13 proteolysis in the presence of 2M urea. The D1653V variant showed only a slight enhancement of proteolysis, indicated by a larger loss of only high MW multimers, in comparison to the WT FLVWF.

### Development of a VWF A2 FRET construct

To determine the suitability of the isolated VWF A2 domain as a model to study these VWF A2 variants, they were introduced into the VWF A2 domain expression plasmid. Following transient transfection in HEK293-EBNA cells, the majority of the type 2A variants were secreted (S1 Fig). Upon purification, the mutated recombinant VWF A2 domain fragments appeared to aggregate, preventing viable functional studies of domain unfolding. The aggregation of the variants could be overcome by incorporation into the larger VWF A1A2A3 construct. It was anticipated that fusing fluorescent proteins to the N- and C-termini of the VWF A2 domain (in essence replacing the VWF A1 and A3 domains of the VWF A1A2A3 fragment) may overcome the aggregation problem observed upon the purification of VWF A2 domain Type 2A mutants.

**Fig 2:**
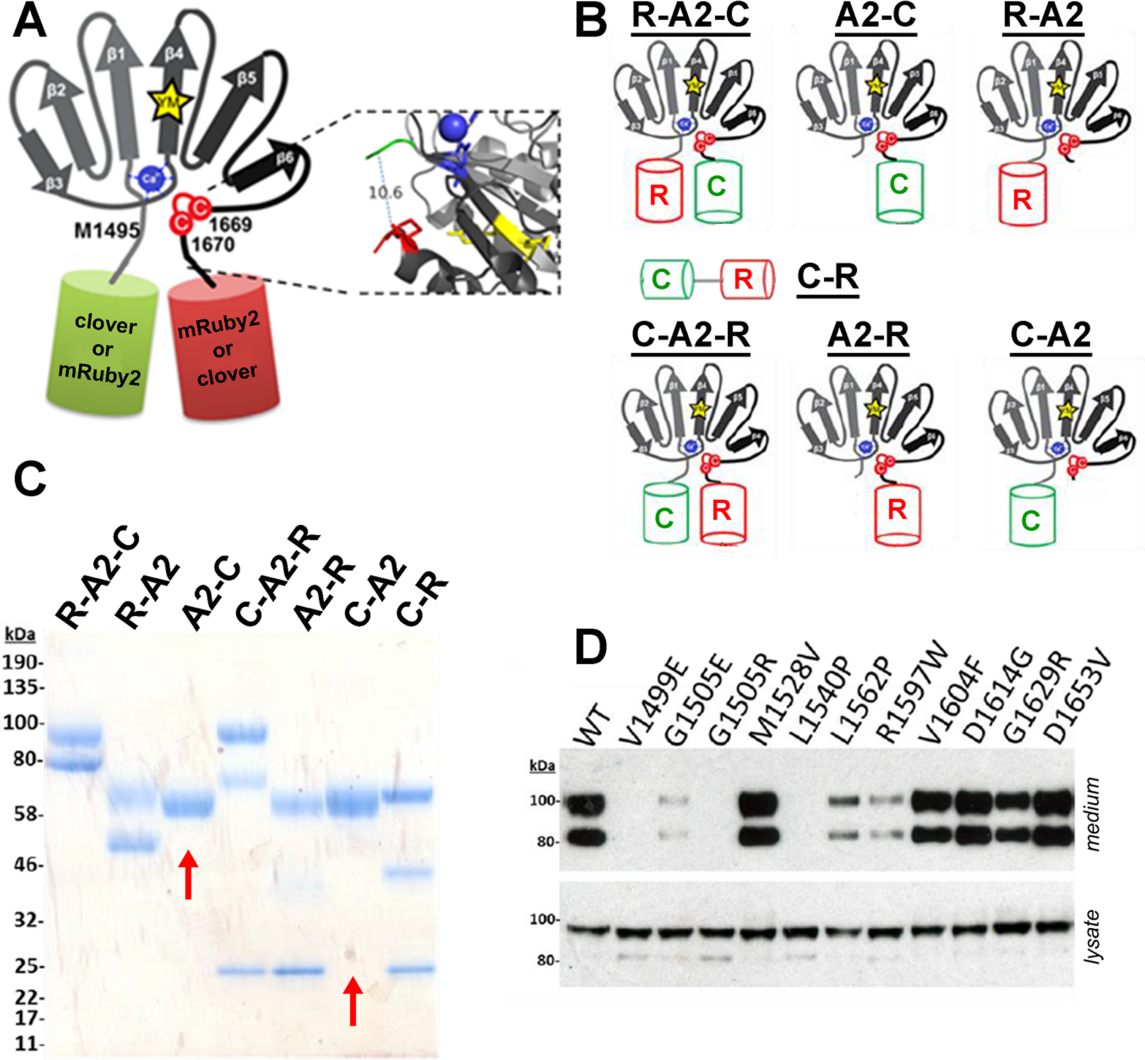
VWF A2 FRET and VWD variants. (A) Cartoon representation of the VWF A2 domain structure, the N-terminal (M1495) fused to clover (or mRuby2) and C-terminal (C1670) fused to a mRuby2 (or clover). A distance of 10.6Å between M1495 (green) and C1670 (red) was demonstrated in the crystal structure of the VWF A2 domain (3ZQK) using PYMOL. (B) Representation of the VWF A2 FRET constructs with mRuby2 positioned at the N-terminus and clover at the C-terminus (R-A2-C), deletion of mRuby2 from the R-A2-C construct (A2-C), deletion of clover from the R-A2-C construct (R-A2), clover positioned at the N-terminus and mRuby2 at the C-terminus (C-A2-R), deletion of clover from the C-A2-R construct (A2-R), deletion of mRuby2 from the C-A2-R construct (C-A2) and replacement of the VWF A2 domain with a short six amino acid linked, PKRNSG (C-R). (C) Expressed and purified protein samples were analysed by SDS-PAGE and Coomassie stained; red arrows indicate the absence of a fragmentation product. (D) The R-A2-C type 2A VWD variants were transiently transfected in HEK293-EBNA cells. After 3–5 days, the media was collected, cells washed with PBS and then lysed with 1% ipegal. Media and lysate samples were analsyed by SDS-PAGE followed by western blot using an antibody against the C-terminal Myc-tag.

The VWF A2 domain was predicted to be a suitable FRET candidate due to the proximity (~10Å) of the N- and C- termini observed in the crystal structure, 3ZQK[12] (Fig 2A). The defined FRET pair of clover and mRuby2[34] were cloned into the VWF A2 domain expression plasmid, PCEP-A2VicCC, fusing the fluorescent proteins to the N- and C- termini of the VWF A2 domain. The unfolding changes that precede ADAMTS13 proteolysis were anticipated to cause the N- and C- termini of the VWF A2 domain to move apart (potentially by as much as ~570Å[10]).

Optimal positioning was determined for the FRET constructs: mRuby2 was fused to the N-terminus and clover to the C-terminus (protein termed, R-A2-C) and vice-versa (C-A2-R). To provide a positive FRET control, the VWF A2 domain was replaced with a short six amino acid linker (C-R). To provide negative FRET controls, mRuby2 or clover were removed from the expression plasmids (Fig 2B). After expression of the constructs in HEK293-EBNA cells and purification by Ni^2+^ affinity chromatography, the purified proteins were examined by SDS-PAGE followed by Coomassie staining (Fig 2C). Fragmentation products were detected for the proteins containing mRuby2, but absent within the proteins where mRuby2 had been removed (Fig 2C, red arrows). It has previously been reported that an intrinsic, autocatalytic step in the formation of the mature chromophore results in fragmentation of the protein backbone in RFPs[44–48].

**Fig 3:**
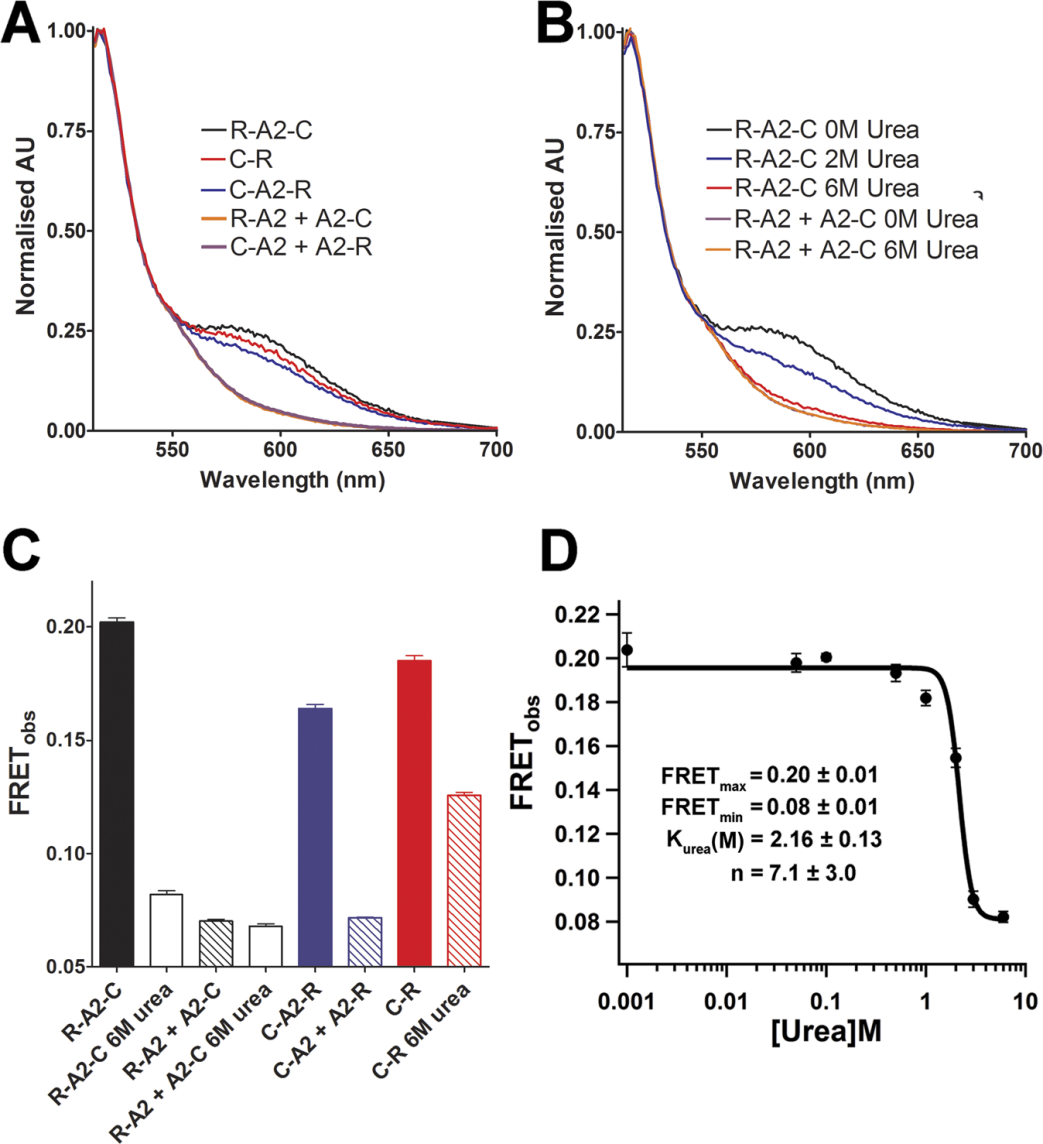
FRET efficiency of VWF A2 FRET proteins. (A) Normalised emission spectra at Ex_505_ for R-A2-C, C-R, C-A2-R, R-A2+A2-C and C-A2+A2-R. (B) Normalised emission spectra at Ex_505_ for R-A2-C and R-A2+A2-C in 0 and 6M urea concentrations. The normalised spectra for R-A2+A2-C (0 and 6M urea) and C-A2+A2-R overlay. Spectra are representative of three separate scans. (C) FRET_obs_ values were determined for each variant(s) using the equation FRET_obs_ = Ex_505_Em_585_/ (Ex_505_Em_585_+ Ex_505_Em_516_). (D) FRET_obs_ values for urea titration against R-A2-C (VWF A2 FRET). The curve was fitted using the Hill titration equation. The fitted curve allows for calculation of the urea concentration at the mid-point of unfolding (K_urea_), the cooperativity of unfolding (Hill coefficent, n) and the maximum and minimum FRET_obs_ values of the fitted curve. Results are the mean±SD of at least 3 separate scans.

To determine whether FRET occurs with the VWF A2 domain constructs, excitation and emission scans of the different purified chimeric proteins were performed. To determine the influence of non-specific clover/mRuby2 interactions in solution on the FRET_obs_, the deletion variants were combined at equimolar concentrations (that is, R-A2+A2-C and C-A2+A2-R). The increase in emission between 550-650nm with excitation at 505nm (Ex_505_) in the single FRET constructs, in contrast to its absence with the combination of deletion variants, indicate that FRET can be observed in the R-A2-C, C-A2-R and C-R constructs (Fig 3A). Additionally, an increase in signal in the excitation spectra between 450-525nm with fluorescent detection at 650nm (Em_650_), further confirmed the observations made with the emission spectra (S2 Fig). A high concentration of urea (6M) was used to induce the unfolding of the VWF A2 domain. This caused a decrease in the FRET_obs_ to that of the deletion variants, indicating the loss of FRET upon urea-induced VWF A2 domain unfolding within the R-A2-C construct (Fig 3B and 3C). No change in the emission spectra of the deletion variants was observed in 6M urea, indicating that the fluorescent proteins were not affected by the high concentration of urea (Fig 3B and 3C). A reduction in FRET_obs_ was observed with the C-R (Fig 3C), but as the emission/excitation spectra of the individual Clover (A2-C) and mRuby2 (R-A2) fluorescent proteins were unaffected by urea (S3 Fig), it was concluded that this was an effect of urea on the relative orientation of these FP in C-R. The R-A2-C construct was chosen for further analysis and the introduction of type 2A variants (Fig 3D), due to it displaying the highest FRET_obs_ (Fig 3C).

Urea titrations were performed to assess the influence of VWF A2 domain unfolding on the FRET_obs_. The decrease in FRET_obs_ with increasing concentrations of urea indicated the sensitivity of the R-A2-C construct for measuring global conformational change in the VWF A2 domain. Fitting of the urea denaturation allowed for the calculation of the urea concentration of the mid-point of unfolding (K_urea_) and the cooperativity of unfolding (n), as well as FRET_max_ and FRET_min_ values of the fitted curve (Fig 3D).

### Disruption of VWF A2 domain conformation and unfolding by VWD mutations

Of the 11 type 2A variants introduced in the R-A2-C (in the following referred to as VWF A2 FRET) construct, three exhibited complete intracellular retention indicative of misfolding (V1499E, G1505R, L1540P), three displayed reduced secretion in comparison to the WT (G1505E, L1562P, R1597W) and five were secreted with similar efficiency as the WT (M1528V, V1604F, D1614G, G1629R, D1653V) (Fig 2D). Purification of the eight secreted VWF A2 FRET variants yielded sufficient monomeric protein for functional analyses by FRET experimentation.

**Fig 4:**
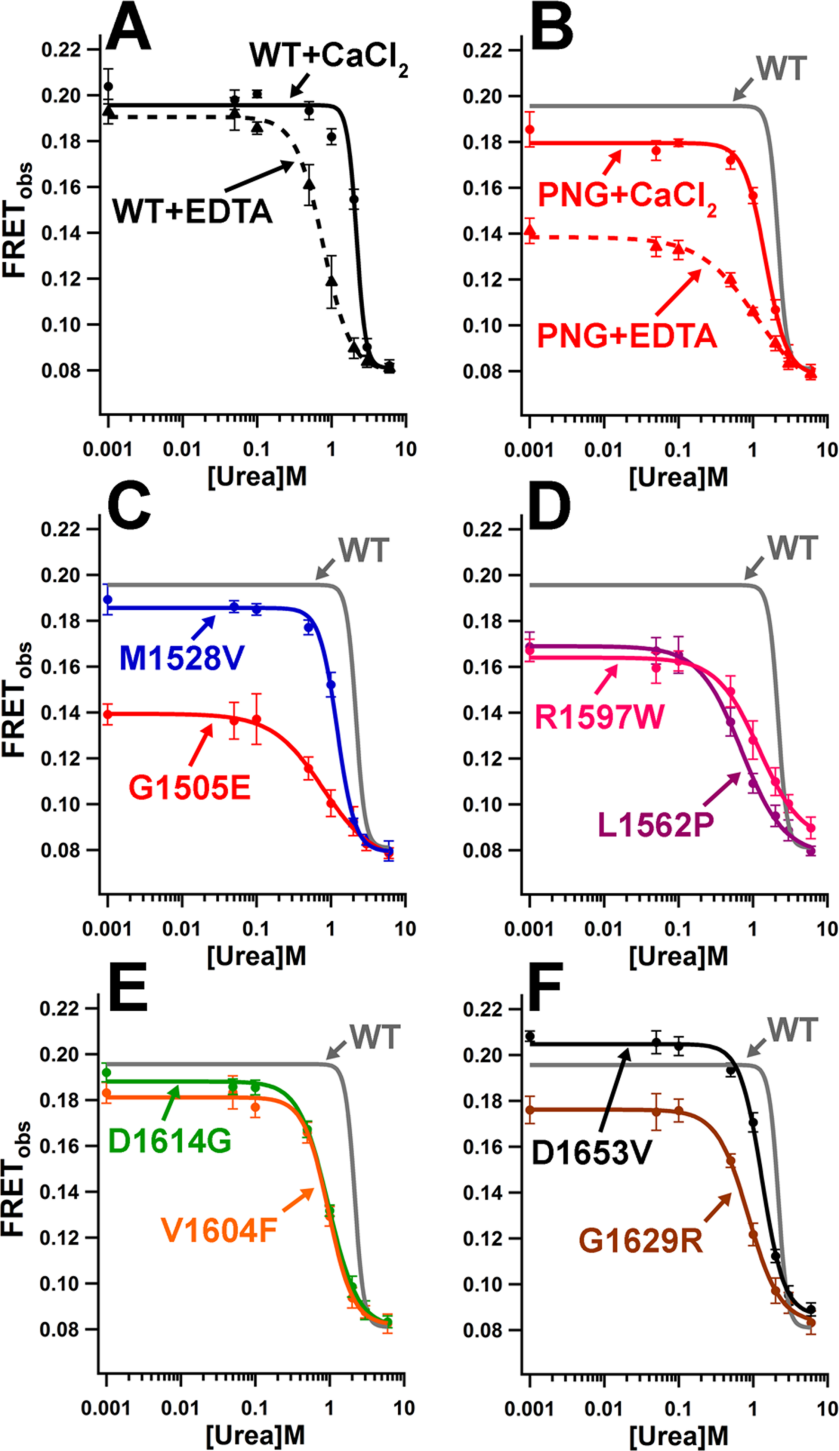
VWD mutations disrupt the cooperativity of unfolding and stability of the VWF A2 domain. (A-B) FRET_obs_ measurements were carried out in the presence of 20mM Tris (pH7.8), 50mM NaCl and 1mM EDTA (triangles, dashed lines) or 1.25mM CaCl_2_ (circles, solid lines) with varying concentrations of urea. WT VWF A2 FRET was studied (black lines) as well as the VWF A2 FRET glycan variant obtained following digestion with PNGase (PNG, red lines) (C-F) FRET_obs_ measurements were carried out exclusively in 20mM Tris (pH7.8), 50mM NaCl, 1.25mM CaCl_2_ with varying concentrations of urea for all VWF A2 FRET type 2A VWD variants (various colours) and the fitted curve for WT is indicated (grey lines). Results are means ±SD of at least 3 separate scans.

Control experiments were performed using EDTA to chelate the VWF A2 stabilising Ca^2+^ ion, and PNGase treatment to remove the stabilising N-linked glycan from the VWF A2 domain. Selective disruption of Ca^2+^ binding (WT+EDTA) and removal of N-linked glycans (PNGase+CaCl_2_) caused a shift in K_urea_ of -1.40M and -0.70M in comparison to the WT (2.16±0.13M), indicating a reduction in folding stability (Fig 4A and 4B, Table 2). Surprisingly, the combined removal of Ca^2+^ and glycans from the WT VWF A2 FRET protein (PNGase+EDTA) resulted in a higher K_urea_ value (1.11±0.24) than WT+EDTA (0.77±0.03). The lower FRET_max_ and n values for PNGase+EDTA, in comparison to WT+EDTA and WT+CaCl_2_ (Table 2), indicates a partial unfolding in the PNGase+EDTA variant in the absence of urea and a less cooperative, shortened transition to the fully unfolded state (FRET_min_). Collectively, the decrease in FRET_max_, K_urea_ and n in the order WT+CaCl_2_ > PNGase+CaCl_2_ > WT+EDTA > PNGase+EDTA, is consistent with decrease in thermostability that we previously determined using differential scanning fluorimetry for the condition/treatment matched VWF A2 domain fragments[16].

Analysis of the purified type 2A VWF A2 FRET variants showed that all of the mutations, except D1653V, caused a decrease in FRET_max_ relative to the WT, 0.20±0.01 (Table 2). The G1505E variant displayed the largest decrease in FRET_max_, 0.14±0.01 (Fig 4C) and displayed a similar unfolding transition to that of the PNGase+EDTA fragment (Fig 4B). The M1528V variant displayed a smaller decrease in FRET_max_, 0.19±0.01, with a -0.95M shift in K_urea_ indicating a reduction in folding stability in comparison to the WT (Fig 4C, Table 2). The variants L1562P, R1597W, V1604F, D1614G and G1629R all displayed a reduction in folding stability with K_urea_’s of 0.70±0.04M, 1.22±0.16M, 0.96±0.04M, 0.95±0.04M and 0.86±0.03M, respectively, as well as lower cooperativities of unfolding than the WT (Fig 4C–4F, Table 2). Although the D1653V variant displayed a slight increase in FRET_max_ (0.20±0.01), a small reduction folding stability was observed with urea (K_urea_ = 1.32± 0.06M) when compared to the WT (Fig 4F, Table 2). There was a statistically significant difference between the K_urea_ values (Table 2) as determined by one-way ANOVA (P<0.0001, F = 43.63, R^2^ = 0.95). All type 2A variants (plus EDTA and PNGase treatments of the WT) showed a statistically significant reduction in K_urea_ (P<0.01) using posthoc Dunnett's Multiple Comparison Test to WT CaCl_2_ (GraphPad Prism).

### ADAMTS13 proteolysis of VWF A2 FRET variants

**Fig 5:**
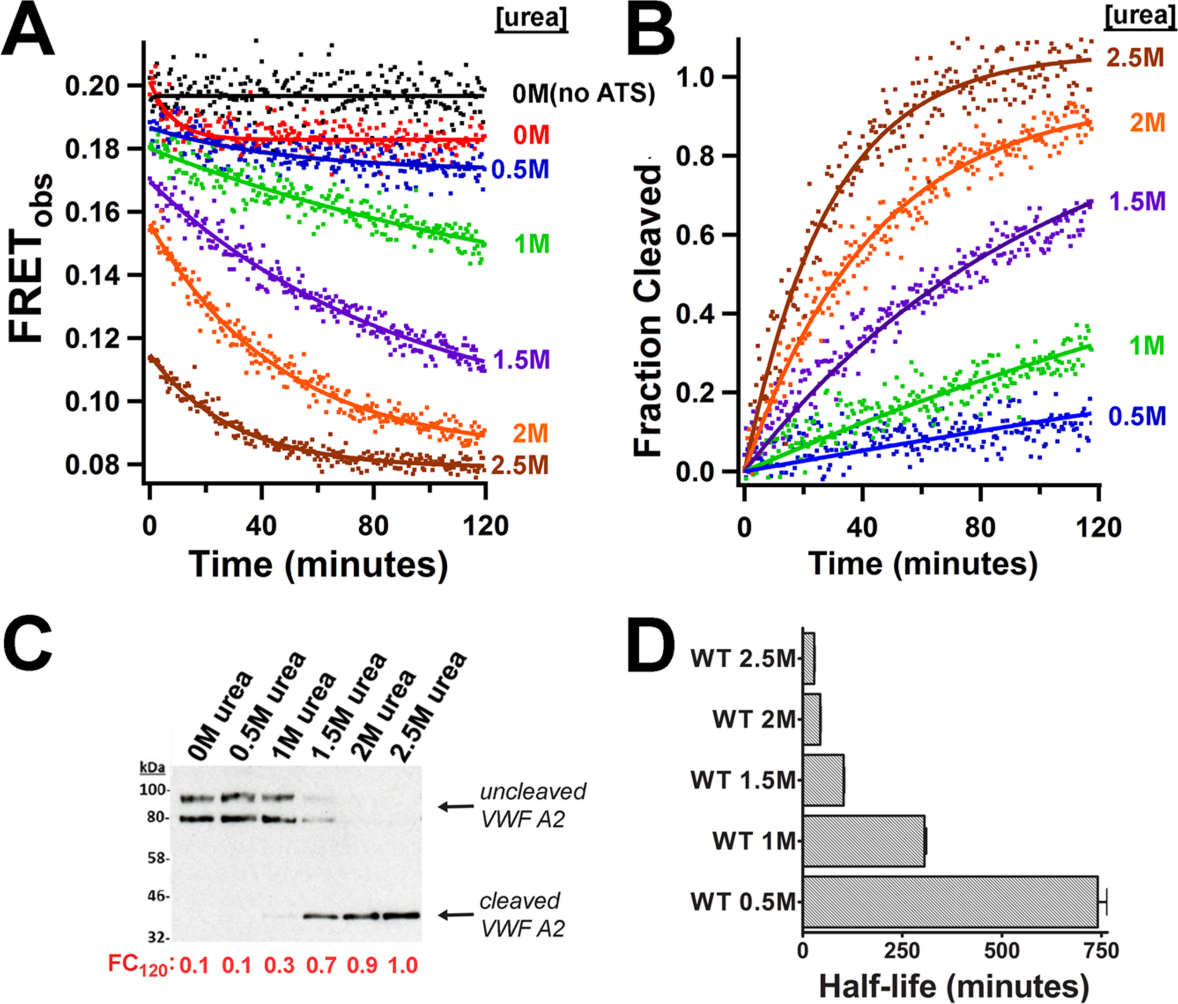
VWF A2 FRET is effectively proteolysed by ADAMTS13. A final concentration 50nM of the WT VWF A2 FRET construct and 50nM ADAMTS13 (or 0nM ADAMTS13 plus 0M urea, termed ‘no ATS’) were separately pre-incubated in 20mM Tris (pH7.8), 50mM NaCl, 1.25mM CaCl_2_ and varying concentrations of urea at 37°C for 45mins before being combined to initiate the proteolysis reaction. (A) Emission scans were taken at Ex_505_ every 0.5mins for 120mins, after 2mins ADAMTS13 was added and FRET_obs_ plotted against time. (B) The FRET_obs_ data was converted to ‘Fraction Cleaved’. (C) After 120mins reactions were stopped by the addition of EDTA followed by a western blot using an anti-Myc antibody. The calculated Fraction Cleaved after 120 minutes (FC_120_) is appended in red. (D) The half-life of proteolysis of the WT in differing concentrations of urea by 50nM ADAMTS13 was calculated by fitting the Fraction Cleaved data with an exponential rise function.

ADAMTS13 proteolysis assays were performed with WT VWF A2 FRET protein in increasing concentrations of urea, with FRET_obs_ measurements taken every 0.5 min for 120 mins. A decrease in FRET_obs_ during the incubation with ADAMTS13 was demonstrated, with larger decreases with higher urea concentrations (Fig 5A). The FRET_obs_ data was converted to Fraction Cleaved (FC). The calculated FC values after a 120-minute incubation with ADAMTS13 (FC_120_) for the WT VWF A2 FRET are consistent with the western blot analysis of the same samples (Fig 5B and 5C). *In situ* measurements of the proteolysis reaction allowed for calucation of the half-life of proteolysis of VWF A2 FRET (Fig 5D). For the WT, a very long half-life is calculated in 0M and 0.5M indicating that the cleavage occurs very slowly, if at all. Upon addition of increasing concentrations of urea, the rate of ADAMTS13 proteolysis increased, with the half lives of 43.6±1.2mins and 28.6±0.9mins in 2M and 2.5M urea, respectively. Proteolysis assays were also carried out with a WT A1A2A3 fragment using similar protein concentrations. Similarly to VWF A2 FRET, cleavage products were only observed at concentrations of urea above 1.5M urea, but, no half life could be calculated. These results suggest that the VWF A2 FRET construct behaves similarly to the WT VWF A1A2A3 fragment with respect to its interaction with ADAMTS13.

**Fig 6:**
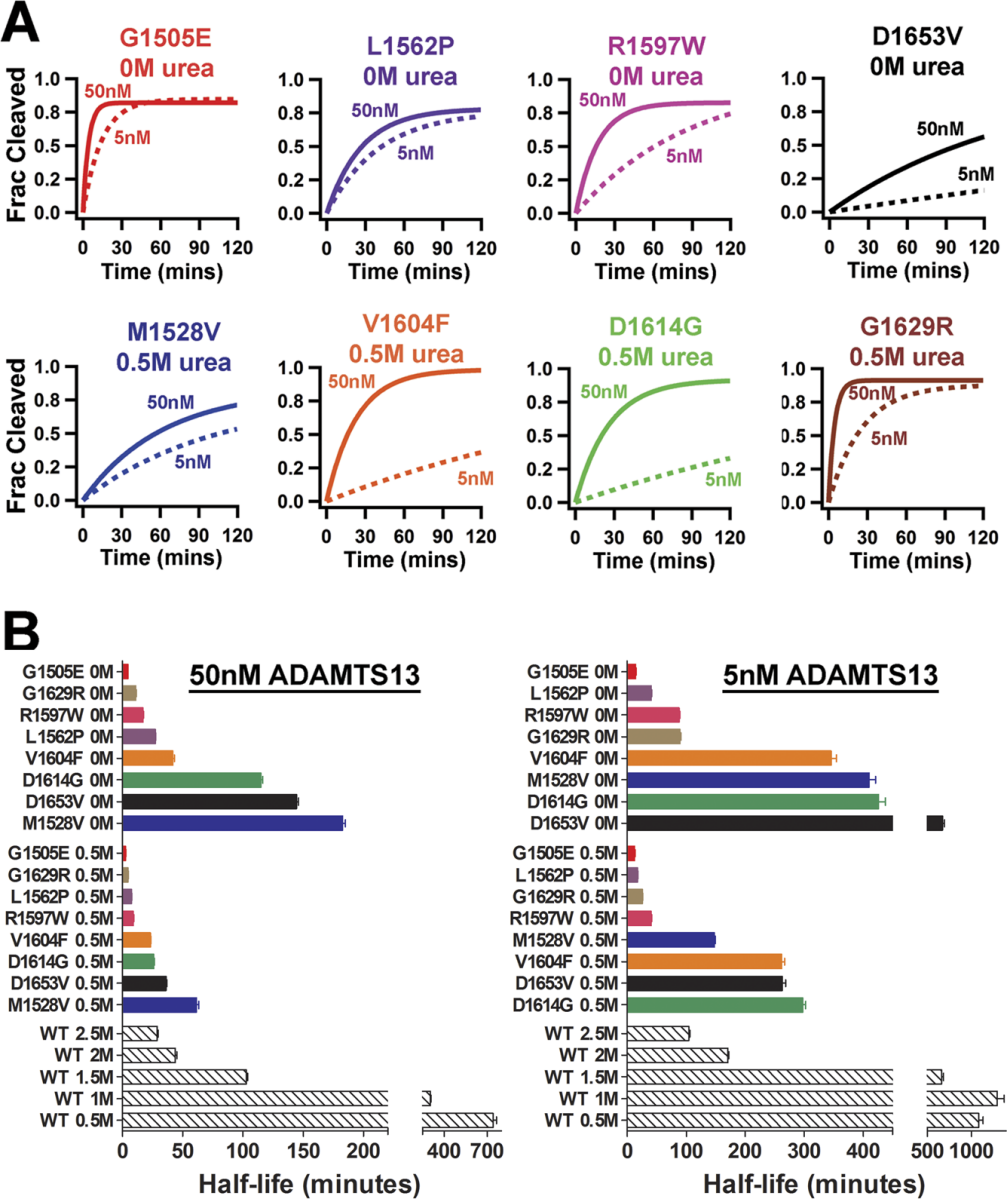
Type 2A VWD variants enhance the rate of ADAMTS13 proteolysis. VWF A2 FRET variants (50nM) and 50nM ADAMTS13 or 5nM ADAMTS13 were separately pre-incubated in 1.25mM CaCl_2_ ± 0.5M urea for at 37°C for 45mins before being combined to initiate proteolysis. Emission scans were taken at Ex_505_ every 0.5mins for 120mins, after 2mins ADAMTS13 was added. FRET_obs_ data was converted to Fraction Cleaved (Frac Cleaved). (A) Selected exponential rise fits are shown for type 2A VWD variants with either 0M (upper panel) or 0.5M urea (lower panel) and 50nM (solid lines) and 5nM (dashed lines) ADAMTS13. (B) Half-lives were calculated and are displayed for type 2A VWD variants (grouped by 0M and 0.5M urea in ascending order, various coloured bars) and WT (0.5M, 1M, 1.5M, 2M and 2.5M urea, black diagonal lined bars) in ascending order.

Next, time course assays with the type 2A VWF A2 FRET variants were performed with either 50nM or 5nM ADAMTS13 ±0.5M urea. As expected, all of the type 2A variants displayed enhanced proteolysis, with an increase in the rate of proteolysis (i.e. reduced half-lives) with 50nM ADAMTS13 in comparison to 5nM ADAMTS13 (Fig 6). Notably, the V1604F and D1614G variants had much shorter half-lives in 50nM ADAMTS13 than 5nM ADAMTS13 (Fig 6A, lower panel). This disparity that may be explained by the location of the V1604F[49, 50] and D1614G[51] mutations in known ADAMTS13 docking sites. Overall, it was found that all of the type 2A VWF A2 FRET variants tested exhibited enhanced proteolysis by ADAMTS13, regardless of their location within ADAMTS13 binding sites, with the half-lives displaying a large range: varying from 2.5±0.3mins (G1505E 0.5M urea, 50nM ADAMTS13) to 672.9±25.6mins (D1653V 0M urea, 5nM ADAMTS13) (Fig 6B).

## Discussion

The sequencing of the *VWF* gene in patients with a bleeding phenotype has been informative for understanding of the function of VWF and its domains since the 1990s[52]. Over 30 point mutations in the VWF A2 domain have been reported in patients in association with the loss of HMW multimers of VWF, consistent with the type 2A VWD phenotype. Previous studies have shown that three of these VWF A2 domain mutations (M1528V, R1597W and E1638K) reduce the thermostability of the purified VWF A2 domain[25], while the introduction of 11 VWF A2 mutations (G1505E, G1505R, S1506L, M1528V, R1597W, V1607D, G1609R, I1628T, G1629E, G1631D and E1638K) into a VWF A1A2A3 fragment has been shown to result in enhanced proteolysis in comparison to the WT fragment in both the presence and absence of urea [26]. Molecular dynamic simulations of the unfolding of the crystal structure of the VWF A2 domain (3GXB, a structure that does not, however, contain the bound calcium ion), have indicated that VWF A2 domain mutations may disrupt the unfolding of the VWF A2 domain[31–33]. Molecular dynamic simulations (in which mutations were introduced into the 3GXB structure) further predict that type 2A mutations L1567I, I1628K and E1638K reduce the force required for the domain to unfold [29], also, that the G1629E variant thermodynamically destabilises the domain[30]. Pre-2009 homology-based models of the VWF A2 domain[27, 28], show by molecular dynamic simulation and free energy calculations that L1503Q, G1505E, G1505R, S1506L, L1540P, R1597W, I1628T, and E1638K disrupt the fold of the VWF A2 domain[28]. The structures, single molecule pulling experiments and molecular dynamic simulations of the WT VWF A2 domain predict that unfolding occurs by forced separation of the N- and C- termini[10–13, 15].

To understand better the mechanism of mutations in the VWF A2 domain, we developed a VWF A2 FRET construct. Fusion of mRuby2 and clover to the N- and C- termini of the VWF A2 domain prevented aggregation and allowed the measurement of FRET between the termini and their response to urea-induced unfolding and proteolysis. Urea-induced unfolding experiments with the VWF A2 FRET construct confirmed that EDTA and PNGase treatment destabilise the VWF A2 domain, consistent with previous results using differential scanning fluorimetry (DSF)[12, 16, 17] and circular dichroism (CD)[15]. The cleavage of the WT VWF A2 FRET protein by ADAMTS13 in concentrations of urea >1.5M (Fig 5) is consistent with multiple studies of recombinant WT VWF fragments containing the VWF A2 domain[12, 13, 26, 29]. This designed FRET system allows the study of multiple VWF and ADAMTS13 variants under varying conditions, a distinct advantage over alternative techniques for evaluation of VWF A2 unfolding. The folding stability (K_urea_), the cooperativity of unfolding (n), *in situ* measurements allow calculation of the half-life of ADAMTS13 proteolysis and the relative proximity of the N- and C- termini of the VWF A2 domain in the absence of denaturant (see below) can be determined.

A representative panel of 10 mutations in the VWF A2 domain were chosen from the ISTH-SSC VWF database (V1499E, G1505E, G1505R, M1528V, L1540P, L1562P, R1597W, V1604F, D1614G, G1629R), together with a novel unreported variant (D1653V), (Table 1). All mutations had been reported to be associated with a type 2A VWD phenotype, although VWF levels were not available in four of the VWF A2 variants selected (M1528V, L1562P, V1604F and D1614G). Initially, to define the mechanism of multimer loss, all 11 FLVWF VWF A2 variants were transiently expressed in HEK293 cells. Three of the variants (V1499E, G1505R and L1540P) displayed reduced secretion (Table 1), consistent with the type 2A group 1 VWD mechanism [18, 19]. ADAMTS13 proteolysis of the secreted FLVWF variants indicated that all of the variants displayed enhanced susceptibility to proteolysis, with a range of severities in comparison to the WT (Fig 1), consistent with type 2A group 2 VWD [18, 19].

To further investigate the underlying mechanism of the enhanced ADAMTS13 proteolysis, the 11 A2 domain variants were introduced into the VWF A2 FRET construct. As was seen with the expression of FLVWF, the V1499E, G1505R and L1540P variants displayed a much reduced secretion with intracellular retention, indicative of misfolding[53] (Fig 2D). All of the secreted and purified VWF A2 FRET variants displayed a reduction in folding stability (K_urea_) and unfolding cooperativity (n) in comparison to the WT (Fig 4, Table 2). Again, similarly to the ADAMTS13 proteolysis of FLVWF (Fig 1), a range in severity is determined across the VWF A2 variants.

**Fig 7:**
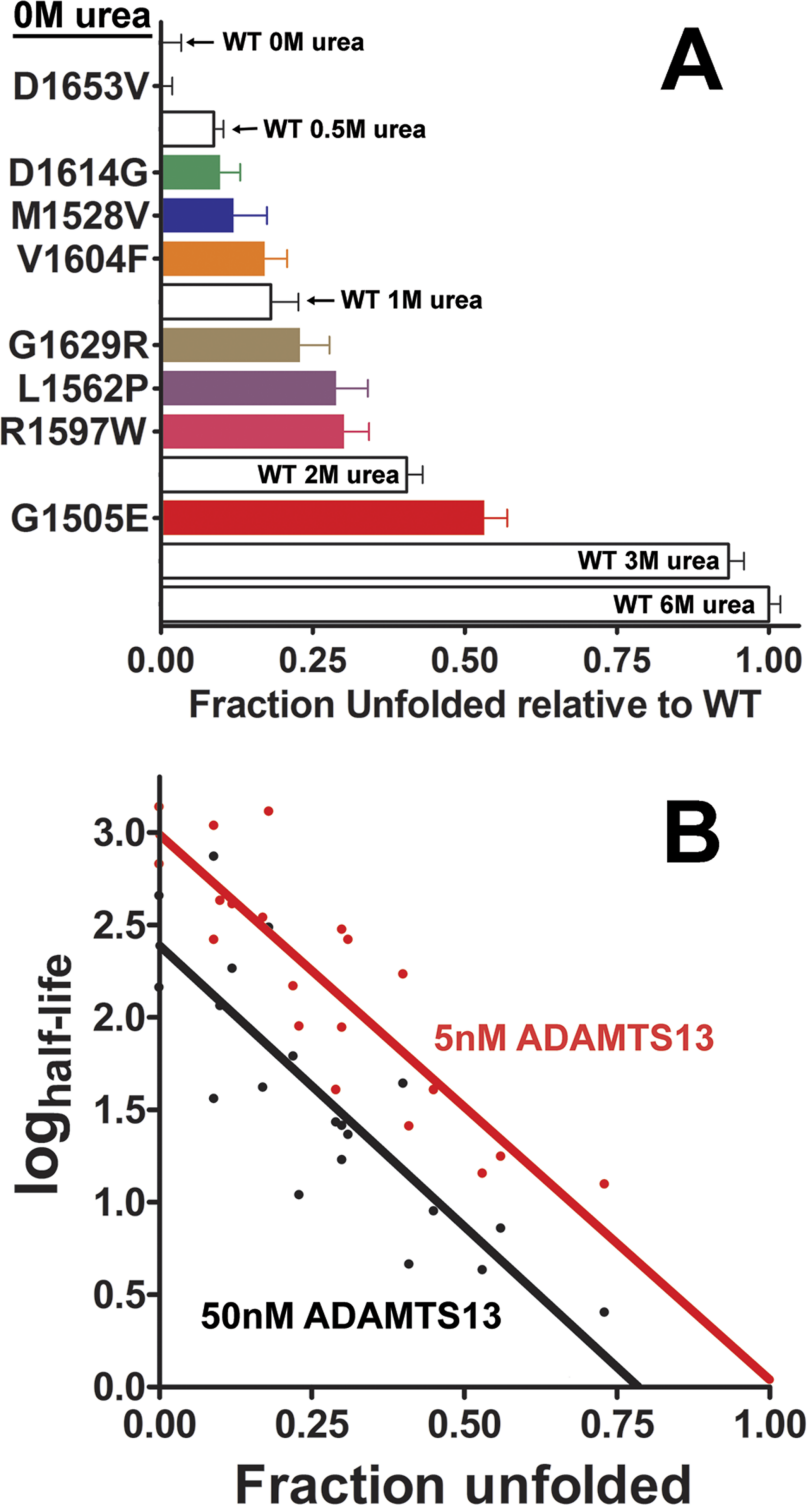
Type 2A VWD mutations enhance unfolding of the VWF A2 domain accelerating ADAMTS13 proteolysis. (A) FRET_obs_ values for the VWD type 2A variants in 0M urea were converted to ‘Fraction unfolded’ using the assumption that the WT VWF A2 (FRET) domain is fully folded in 0M urea and fully unfolded in 6M urea. (B) The calculated log of the half-time of ADAMTS13 proteolysis for type 2A VWD variants and WT (Fig 6B) were plotted against the variant and condition matched Fraction unfolded values. A linear relationship between the log half-time values and Fraction Unfolded is indicated for both 5nM ADAMTS13 (red, Spearman coefficient, r^2^ = 0.77) and 50nM ADAMTS13 (black, r^2^ = 0.72).

Interestingly, a reduction in FRET_max_ occurred for the majority of the VWF A2 variants (Table 2 and Fig 4). If the assumption is made that the VWF A2 domain is fully folded in WT VWF A2 FRET 0M urea (WT FRET_max_, 0) and fully unfolded in 6M urea (FRET_min_, 1), the ‘Fraction unfolded relative to WT’ can be calculated from the FRET_obs_ values. Thus, the reduction in FRET_max_ of the VWF A2 variants (in the absence of induced unfolding, i.e. 0M urea) can be interpreted as an induced unfolding that results from the VWD mutations within the VWF A2 domain structure (Fig 7A). To assess the role of VWF A2 domain conformation on the rate of proteolysis with ADAMTS13 (Fig 6), the mutation and urea concentration matched ‘Fraction unfolded’ was plotted against ‘half-life’ of ADAMTS13 proteolysis (Fig 7B). This revealed a logarithmic increase in the rate of proteolysis relative to the unfolded conformation of the VWF A2 domain, for both 5nM and 50nM ADAMTS13. The effects of differing concentrations of urea on ADAMTS13 function and the location of VWF A2 variants in known ADAMTS13 binding sites (V1604F and D1614G), may skew a definitive correlation between unfolding state and rate of proteolysis (Fig 7B).

**Fig 8:**
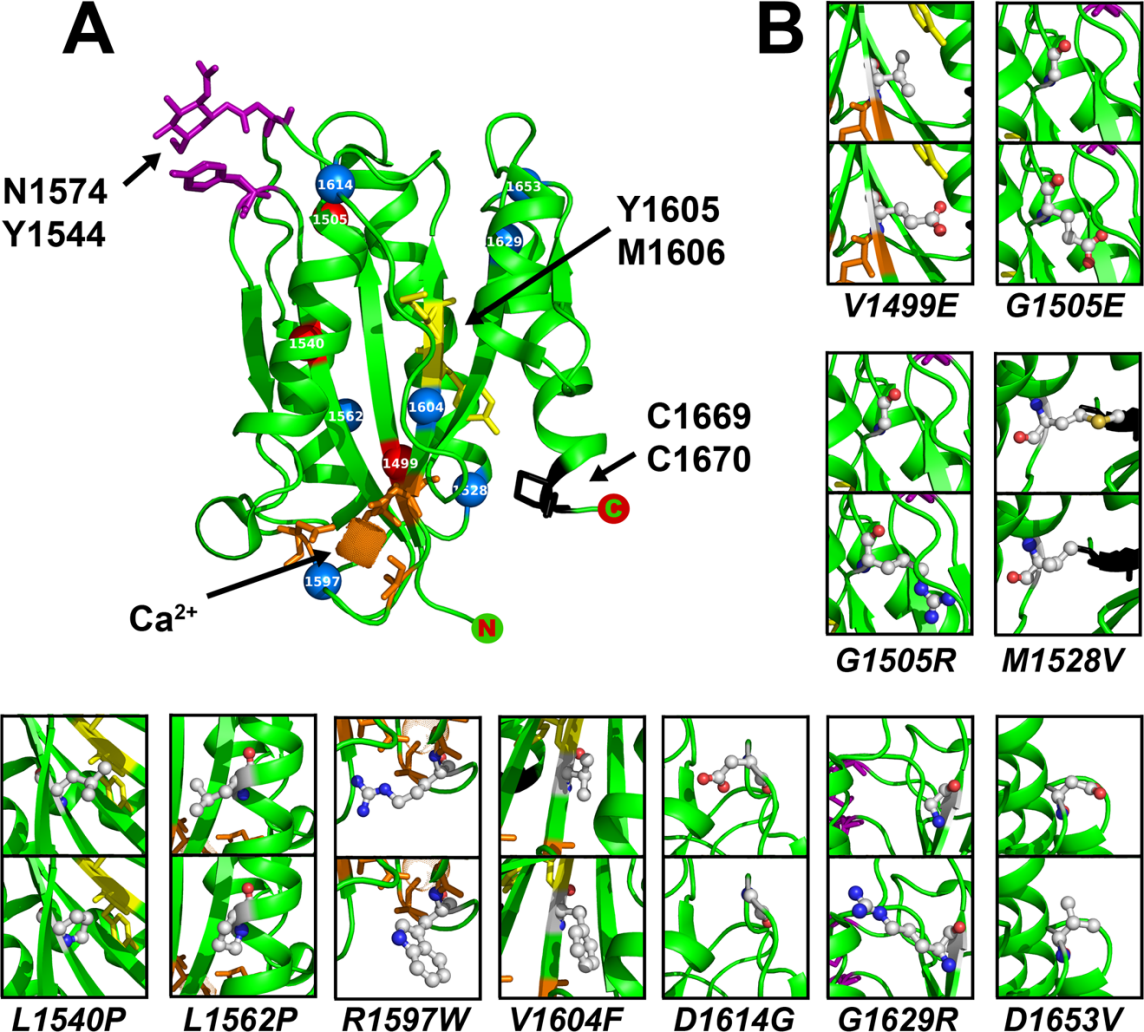
Type2A mutations within the VWF A2 domain. (A) The VWF A2 domain structure (3ZQK) is shown with the stabilising N1574-glycan attachment site (and interacting Y1544 residue, purple), calcium binding site (Ca^2+^ coordinated by D1498, D1596 and N1602A, orange), and vicinal disulphide bond (C1669-C1670, black) plus the scissile bond (Y1605-M1606, yellow) shown with sticks and labelled. The Cα of the location of the type 2A VWD mutations is labelled with a sphere and coloured red for variants that result in increased intracellular retention (V1499E, G1505R and L1540P) and blue for variants that do not effect secretion (G1505E, M1528V, L1562P, R1597W, V1604F, D1614G, G1629R and D1653V). (B) The structure of the amino acids in the WT structure (upper panels) and change caused by mutation in type 2A VWD (lower panels) are shown in ball and stick format: carbon residues are indicated in grey, nitrogen in red, oxygen in blue and sulphur in yellow.

To ascertain if there is a correlation between the location and/or amino acid change of the type 2A mutations with their effect on VWF function, the 11 mutations studied here were mapped on the structure of the VWF A2 domain (Fig 8). The location within secondary structure elements and the accessible surface area (ASA) or solvent accessibility was scored [54], along with the effect of the mutation of FLVWF secretion and the measured VWF A2 FRET parameters (Table 3). Based on this information, no obvious correlation is seen between the location and/or the amino acid change on VWF function. For example, the G1505R variant causes a reduction in secretion in VWF (medium: lysate ratio 0.2), and the G1505E variant causes minimal effect on the secretion of FLVWF (medium: lysate ratio 0.9). Furthermore, there appears to be no clustering of the type 2A mutations within a specific region of the VWF A2 domain (Fig 8), as is observed with VWD type 2B mutations within the VWF A1 domain[55]. As the N- and C- termini of VWF A2 domain are not rigidly held together by a disulphide bond (present in the VWF A1 and A3 domains) any local structural destabilisation caused by an amino acid change is likely to propagate through the domain, causing destabilisation and unfolding. Mutations associated with type 2A VWF group 1 phenotype (V1499E, G1505R and L1540P) are likely to cause the largest destabilisation, resulting in increased intracellular retention of VWF[56].

**Table 3:** Summary of location and functional properties of type 2A VWD mutations in the VWF A2 domain.

Mutation	Position within VWF A2	ASA score	FLVWF	VWF A2 FRET								
			Ratio medium:lysate	K_urea_ (M)			FracU_0_			t_1/2_ (50nM ADAMTS13)		
**V1499E**	β1 sheet	0.00	0.2	n/a			n/a			n/a		
**G1505E**	loop β1-α1	0.00	0.9	0.78	±	0.11	0.53	±	0.04	4.27	±	0.20
**G1505R**	loop β1-α1	0.00	0.2	n/a			n/a			n/a		
**M1528V**	loop α1-β2	0.02	0.9	1.21	±	0.05	0.12	±	0.05	182.57	±	2.34
**L1540P**	β2 sheet	0.00	0.1	n/a			n/a			n/a		
**L1562P**	α2 helix	0.06	1.0	0.70	±	0.04	0.29	±	0.05	26.91	±	0.78
**R1597W**	loop α3-β4	0.23	1.0	1.22	±	0.16	0.30	±	0.04	16.87	±	0.41
**V1604F**	β4 sheet	0.00	1.1	0.96	±	0.04	0.17	±	0.04	41.60	±	1.28
**D1614G**	loop β4-β5	0.24	1.1	0.95	±	0.04	0.10	±	0.03	114.80	±	1.46
**G1629R**	β5 sheet	0.03	1.5	0.86	±	0.03	0.23	±	0.05	10.91	±	0.35
**D1653V**	loop β6-α6	0.32	1.3	1.32	±	0.06	-0.04	±	0.02	144.46	±	1.68

Accessible surface area (ASA) scored using: http://www.abren.net/asaview/ [54]. Residues are scored from 0 (fully buried) to 1.

It can be concluded that regardless of their location within the VWF A2 domain the subset of VWD mutations that do not result in the intracellular retention of VWF (type 2A VWD group 2) disrupt the domain folding stability and cooperativity, causing spatial separation of the N- and C- termini leading to enhanced proteolysis by the regulating protease, ADAMTS13.

## Supporting information

S1 Fig Dot blot analysis of VWF A2 domain expression. The VWF A2 domain (M1574-C1670) VWD Type2A variants were transiently transfected in HEK293 EBNA cells. After 3–5 days, the media was collected, cells washed with PBS and then lysed with 1% ipegal. A 5μL aliquot of media (Med) or lysate (Lys) was dotted onto nitrocellulose membrane and left overnight. After blocking, samples were detected on a western blot using an antibody against the C-terminal Myc-tag.
(PDF)

S2 Fig Emission and excitation spectra of FRET proteins. (A) Normalised emission spectra at Ex475 for 50nM and (B) excitation spectra at Em650 for 250nM R-A2-C, R-A2+A2-C, C-A2-R, C-A2+A2-R and C-R in 20mM Tris pH7.8, 50mM NaCl, 1.25mM CaCl_2_.
(PDF)

S3 Fig Emission and excitation spectra of R-A2 and A2-C. (A+B) Emission and Excitation spectra of 250nM R-A2 in 20mM Tris (pH7.8), 50mM NaCl, 1.25mM CaCl_2_ and 0,2 and 6M urea. (C+D) Emission and Excitation spectra of 50nM A2-C in 20mM Tris (pH7.8), 50mM NaCl, 1.25mM CaCl_2_ and 0,2 and 6M urea.
(PDF)
