# Supplementary material for: A common mechanism by which type 2A von Willebrand disease mutations enhance ADAMTS13 proteolysis revealed with a von Willebrand factor A2 domain FRET construct
Source: PLoS One. 2017 Nov 29;12(11):e0188405. doi: 10.1371/journal.pone.0188405 (PMC5706690; doi:10.1371/journal.pone.0188405)
Supplement: S1 Fig — The VWF A2 domain (M1574-C1670) VWD Type2A variants were transiently transfected in HEK293 EBNA cells. After 3–5 days, the media was collected, cells washed with PBS and then lysed with 1% ipegal. A 5μL aliquot of media (Med) or lysate (Lys) was dotted onto nitrocellulose membrane and left overnight. After blocking, samples were detected on a western blot using an antibody against the C-terminal Myc-tag. (PDF) [file pone.0188405.s001.pdf]

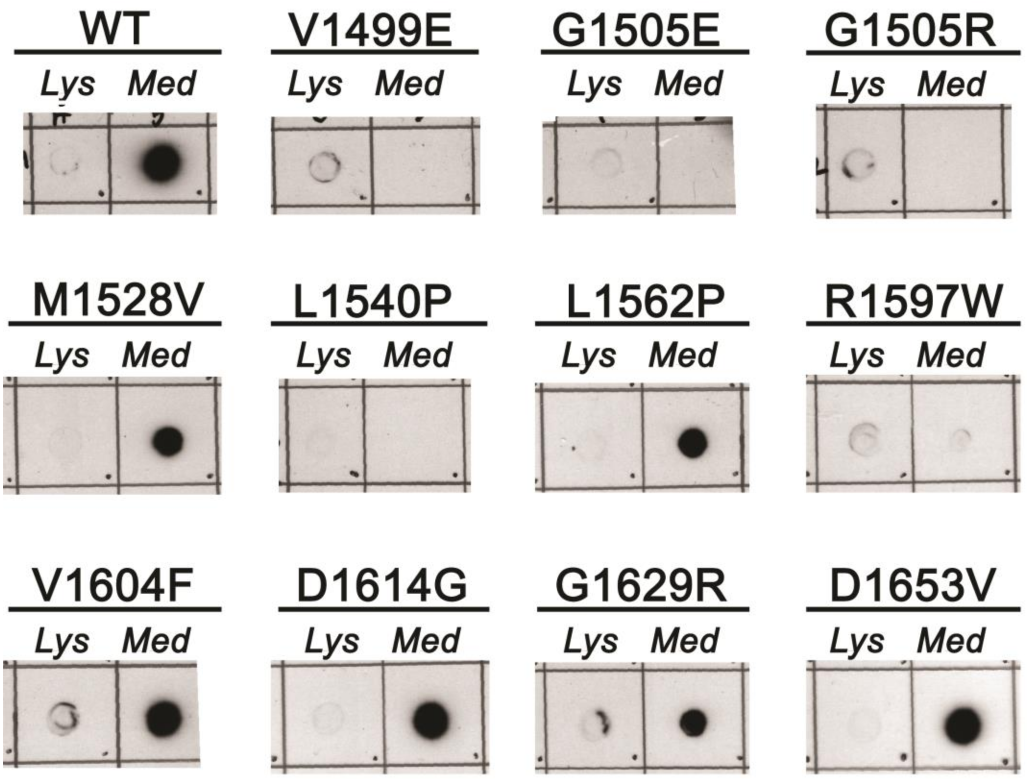

S1 Fig: Dot blot analysis of VWF A2 domain expression

The VWF A2 domain (M1574-C1670) VWD Type2A variants were transiently transfected in HEK293 EBNA cells. After 3-5 days, the media was collected, cells washed with PBS and then lysed with 1% ipegal. A 5µL aliquot of media (Med) or lysate (Lys) was dotted onto nitrocellulose membrane and left overnight. After blocking, samples were detected on a western blot using an antibody against the C-terminal Myc-tag.
