# Supplementary material for: A common mechanism by which type 2A von Willebrand disease mutations enhance ADAMTS13 proteolysis revealed with a von Willebrand factor A2 domain FRET construct
Source: PLoS One. 2017 Nov 29;12(11):e0188405. doi: 10.1371/journal.pone.0188405 (PMC5706690; doi:10.1371/journal.pone.0188405)
Supplement: S2 Fig — (A) Normalised emission spectra at Ex475 for 50nM and (B) excitation spectra at Em650 for 250nM R-A2-C, R-A2+A2-C, C-A2-R, C-A2+A2-R and C-R in 20mM Tris pH7.8, 50mM NaCl, 1.25mM CaCl2. (PDF) [file pone.0188405.s002.pdf]

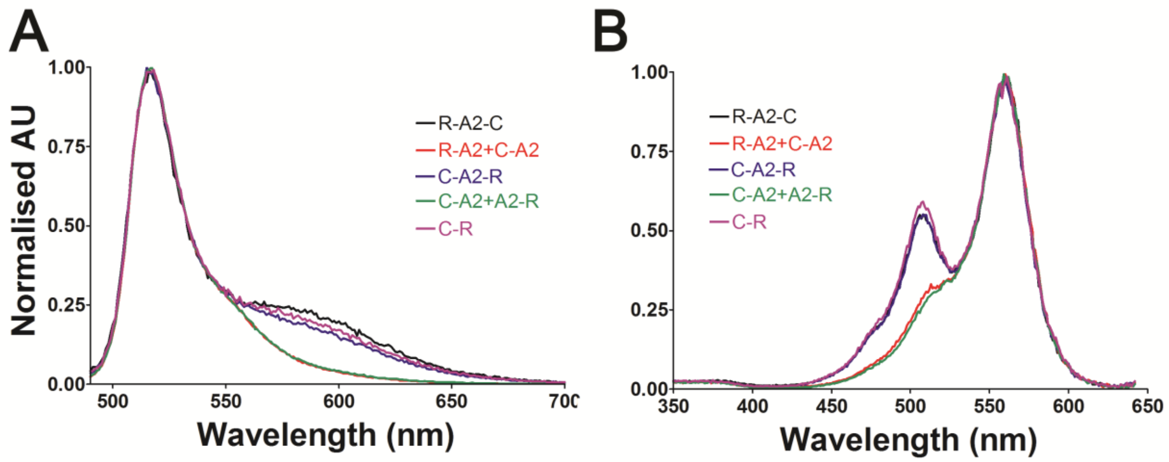

S2 Fig: Emission and Excitation spectra of FRET proteins

(A) Normalised emission spectra at Ex475 for 50nM and (B) excitation spectra at Em650 for 250nM R-A2-C, R-A2+A2-C, C-A2-R, C-A2+A2-R and C-R in 20mM Tris pH7.8, 50mM NaCl, 1.25mM $CaCl_2$.
