# Supplementary material for: A common mechanism by which type 2A von Willebrand disease mutations enhance ADAMTS13 proteolysis revealed with a von Willebrand factor A2 domain FRET construct
Source: PLoS One. 2017 Nov 29;12(11):e0188405. doi: 10.1371/journal.pone.0188405 (PMC5706690; doi:10.1371/journal.pone.0188405)
Supplement: S3 Fig — (A+B) Emission and Excitation spectra of 250nM R-A2 in 20mM Tris (pH7.8), 50mM NaCl, 1.25mM CaCl2 and 0,2 and 6M urea. (C+D) Emission and Excitation spectra of 50nM A2-C in 20mM Tris (pH7.8), 50mM NaCl, 1.25mM CaCl2 and 0,2 and 6M urea. (PDF) [file pone.0188405.s003.pdf]

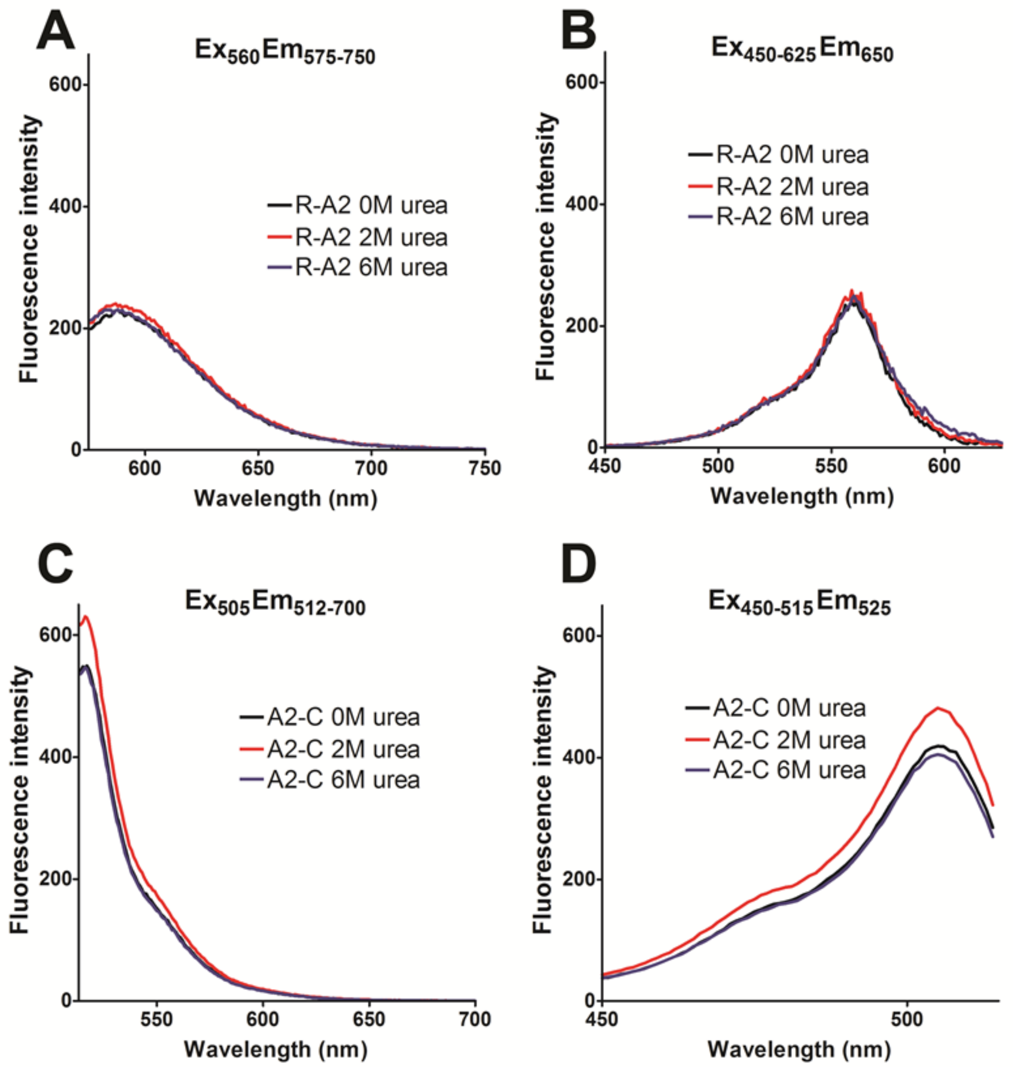

S3 Fig: Emission and Excitation spectra of R-A2 and A2-C

(A+B) Emission and Excitation spectra of 250nM R-A2 in 20mM Tris (pH7.8), 50mM NaCl, 1.25mM $CaCl_2$ and 0,2 and 6M urea. (C+D) Emission and Excitation spectra of 50nM A2-C in 20mM Tris (pH7.8), 50mM NaCl, 1.25mM $CaCl_2$ and 0,2 and 6M urea.
